# The Hospital World

**Published:** 1910-03-19

**Authors:** 


### The Hospital World.

### Personalia.

General Lee has been re-elected chairman of the Cardiff Infirmary.

Dr. Allport, hon. ophthalmic surgeon to the Warneford Hospital, has resigned.

Mr. J. H. Brammall has been re-elected treasurer to the Sheffield Royal Infirmary.

Mr. W. G. Player has been elected president of the Nottingham General Dispensary.

Mr. J. M. Moore has been appointed President of the Ingham Infirmary, South Shields.

Mr. Ludovic Goetz has been elected a member of the House Committee of the London Hospital.

Mr. F. G. Smart, J.P., has been re-elected President of the Tunbridge Wells Homœopathic Hospital.

Mr. S. W. Cocks has been elected chairman of the Board of Management of Urmston Cottage Hospital.

Dr. Allfrey has been elected consulting physician to the Hastings, St. Leonards, and East Sussex Hospital.

Lord Dartmouth has been reappointed President of the Wolverhampton and Staffordshire General Hospital.

Lord Ashburnham has been elected a vice-president of the Hastings, St. Leonards, and East Sussex Hospital.

The Marquis of Winchester has been re-elected President of the Royal South Hants and Southampton Hospital.

Mr. J. R. Wheldon, who has been Secretary to the Ingham Infirmary at South Shields for 19 years, has resigned his post.

Mr. David Davies, M.P., has been elected President of the Montgomeryshire Infirmary in succession to Lord Sudeley.

Mr. T. Aitken has been elected president, Mr. G. Smith hon. treasurer, and Mr. W. Barlow hon. secretary of the Ramsbottom Cottage Hospital.

Prince Francis of Teck, one of the Vice-Presidents of the Middlesex Hospital, has been appointed Chairman of the weekly Board of Governors.

Mr. Frederick Shann has resigned from the honorary medical staff of York County Hospital, and has been appointed honorary consulting surgeon.

Mr. Anderson, hon. secretary, and Mr. Rawlins, hon. treasurer to the Cirencester Cottage Hospital, have been reappointed to their offices for the coming year.

Mr. G. D. Ker has resigned the Treasurership of the Royal Victoria Hospital, Bournemouth, owing to his leaving the town. Sir Gordon Voules has consented to act in his place.

Viscount Clifden has been re-elected president, Mr. C. Trafford hon. treasurer, and Mr. G. P. Paige hon. secretary to the West Cornwall Miners' and Women's Hospital.

Sir Ernest Shackleton has been elected an honorary life-governor of the ("Dreadnought") Seamen's Hospital, in recognition of the services which he had rendered to the Society.

Sir Benjamin V. S. Brodie, Bart., has resigned his position on the committee of the Reigate and Redhill Hospital, and Major Kingsley Foster has been re-elected Chairman.

Miss Jasper, until lately matron of the Mold Cottage Hospital, has been appointed matron of the Oswestry and Ellesmere Cottage Hospital, and will take up her duties on April 2 next. She has had considerable experience of hospital work both in England and abroad, and served in South Africa during the whole of the late war.

Mr. J. Oswell Bury has been elected president and Mr. Philip Yorke, Sir R. E. Egerton, K.C.S.I., Mr. John Jones, and Mr. H. Croom-Johnson, vice-presidents of Wrexham Infirmary.

Mr. Gordon Martyr, for five years in the Secretary's office at St. Mary's, has been appointed Secretary, in place of Mr. J. J. Wycliffe Godwin, resigned, to the Royal Victoria Hospital at Bournemouth.

The following gentlemen have been elected governors of the Royal Isle of Wight County Hospital : Colonel Cradock, J.P., Mr. A. Millward, J.P., Rev. G. L. Davey, Mr. M. Maybrick, and Mr. J. G. Pinnock.

Captain L. W. Vaile, for many years president of the Ramsgate General Hospital, has been compelled to resign on account of his health. Mr. Murray Smith has been elected unanimously to succeed him.

Mr. E. Lort Phillips has been re-elected pesident, and Sir Thomas Freake, Bart., and Major L. F. Knollys, C.M.G., vice-presidents of the Dartmouth Cottage Hospital, of which Mr. Binmore has resigned the chairmanship.

The annual report of the Bideford and District Hospital stated that on the retirement of the last matron 120 applicants had been received, Miss Isabel Lennox, from the Royal County Infirmary, Perth, being appointed matron.

Mr. Cecil W. Beloc, Mr. W. H. Greenshade, Canon Griffiths, Mr. Foster G. Robinson, Mr. Mervyn King, Mr. C. B. Have, and the Rev. J. P. Maud have been elected to serve for three years on the committee of the Bristol General Hospital.

Archdeacon Maude, Prebendary Auden, Rev. A. J. Moriarty, D.D., Mr. Benjamin Blower, Colonel F. Robinson, Mr. Morris, and Mr. Peel, have been elected to the Committee of the Shrewsbury Ear, Eye, and Throat Hospital.

Dr. Arthur Blaikie, M.A., M.B., B.C., has retired from the honorary staff of the Oswestry and Ellesmere Cottage Hospital after twenty-one years' service, owing to ill-health. Dr. Blaikie has been a generous friend and benefactor of the institution.

To fill vacancies on the committee of the West Suffolk Hospital, Bury, Colonel Constantine Hervey, Rev. F. E. Horne, Rev. H. R. Yeo, Rev. Lord Manners Hervey, Mr. C. F. Felton, and Mr. F. C. Andrews (Bury) were elected, several gentlemen retiring by rotation.

Mr. I. Robinson was elected President, Mr. W. Smart and Mr. T. Brown Vice-Presidents, Councillor Dean re-elected Hon. Secretary, and Mr. T. Rylatt Treasurer at the annual meeting of the Working Men's Committee of the Hull Victoria Hospital for Sick Children.

Rev. J. H. A. Gibson has resigned the post of honorary secretary to the Brighton, Hove, and Preston Provident Dispensary, a post which he has filled for thirty-one years with much ability and success. Rev. E. H. Rogers has been elected to fill the vacancy caused by this resignation. Messrs. H. J. Taylor, John A. Tannahill, R. S. Murray, and Richard Downie have been appointed new directors, and Mr. Godfrey P. Collins, M.P. for the burgh, has been elected vice-president of the Greenock Infirmary.

---

The House Committee of the London Hospital has recommended the appointment of Dr. Daly as anæsthetist and of Dr. Bligh Wall and Dr. Austin Cooper as assistant anæsthetists. These two latter appointments are new ones, which have been made necessary owing to the increasing amount of surgical work.

